# A Piezoelectric Immunosensor Using Hybrid Self-Assembled Monolayers for Detection of *Schistosoma japonicum*

**DOI:** 10.1371/journal.pone.0030779

**Published:** 2012-01-27

**Authors:** Shiping Wang, Tieqiu Yin, Shaohua Zeng, Hongli Che, Feifei Yang, Xiuchun Chen, Guoli Shen, Zhaoyang Wu

**Affiliations:** 1 Key Laboratory of Schistosomiasis in Hunan, Department of Parasitology, Xiangya Medical College, Central South University, Changsha, China; 2 The Second People's Hospital of Hunan Province and Brains Hospital of Hunan Province, Department of Clinical Laboratory, Changsha, China; 3 State Key Laboratory of Chemo/Biosensing and Chemometrics, College of Chemistry and Chemical Engineering, Hunan University, Changsha, China; University of Manchester, United Kingdom

## Abstract

**Background:**

The parasite *Schistosoma japonicum* causes schistosomiasis disease, which threatens human life and hampers economic and social development in some Asian countries. An important lesson learned from efforts to reduce the occurrence of schistosomiasis is that the diagnostic approach must be altered as further progress is made towards the control and ultimate elimination of the disease.

**Methodology/Principal Findings:**

Using mixed self-assembled monolayer membrane (mixed SAM) technology, a mixture of mercaptopropionic acid (MPA) and mercaptoethanol (ME) was self-assembled on the surface of quartz crystals by gold-sulphur-bonds. Soluble egg antigens (SEA) of *S. japonicum* were then cross-linked to the quartz crystal using a special coupling agent. As compared with the traditional single self-assembled monolayer immobilization method, *S. japonicum* antigen (*Sj*Ag) immobilization using mixed self-assembled monolayers exhibits much greater immunoreactivity. Under optimal experimental conditions, the detection range is 1∶1500 to 1∶60 (infected rabbit serum dilution ratios). We measured several infected rabbit serum samples with varying *S. japonicum* antibody (*Sj*Ab) concentrations using both immunosensor and ELISA techniques and then produced a correlation analysis. The correlation coefficients reached 0.973.

**Conclusions/Significance:**

We have developed a new, simple, sensitive, and reusable piezoelectric immunosensor that directly detects *Sj*Ab in the serum. This method may represent an alternative to the current diagnostic methods for *S. japonicum* infection in the clinical laboratory or for analysis outside the laboratory.

## Introduction

*Schistosome*, a blood-dwelling fluke, causes schistosomiasis. There are three major *schistosome* species, *Schistosoma haematobium*, *Schistosoma mansoni* and *Schistosoma japonicum (S. japonicum)*. In China, schistosomiasis is primarily caused by *S. japonicum* [1]–[3]. This parasite is considered a true zoonosis due to its wide host range, infecting at least 31 species of mammals, including humans [4]. Larval forms can penetrate the skin of people participating in water activities, and these subsequently develop into adult schistosomes and live in the blood vessels of the host. Long-term infections can result in severe lesions, which may cause blockage of the blood-flow and portal hypertension leading to the potential redirection of eggs into the lungs and brain, where they can be deadly [5].

Currently, some immunological detection methodologies, such as enzyme-linked immunosorbent assays (ELISA) [6], indirect haemagglutination assays (IHA) [7], the circumoval precipitin test (COPT) [8], dot immunogold silver staining (dot-IGSS) [9], radio immunoassay, etc., have been generally used for *S. japonicum* infections clinical analyses. Nevertheless, they are often laborious and time-consuming, and need expensive instruments or radioactive chemicals. For *S. japonicum*, it is still significant and necessary to explore some simple, sensitive, and low cost diagnostic methods, and further validation is required for antibody determination [10].

As a new type of high-sensitivity sensor technology, piezoelectric sensing technology has gained recent attention in the immunoassay field [11]–[14]. In 1964, King [15] first reported the piezoelectric crystal as a component of the gas detection hygrometer. A great amount of work by many researchers was subsequently conducted to analyze and measure the gas environment with the piezoelectric crystal. Many gases, including SO_2_, CO, HCl and volatile aromatic substances, were detected; however, bio-sensitive membranes were not used. In 1972, Shons, et al. tested the antibody activity of liquor with the piezoelectric quartz crystal [16]. Since the 1980s, the piezoelectric crystal has been successfully applied for liquor oscillation. The resonant frequency of an oscillating piezoelectric crystal can be affected by a change in the mass at its surface. If the crystal surface is coated with an antigen, the binding of specific antibody to that antigen and the consequent increase in mass on that surface can be detected by a change in frequency. Piezoelectric immunoassay has been applied extensively in environmental and food-related fields and clinical laboratories [17]–[21].

The piezoelectric immunosensor, a type of biological sensor device, combined with high sensitivity and specificity of the immune response of the weight of matter exhibited by quartz crystal, has many advantages for biological and chemical testing. Mercapto self-assembled monolayer membranes (SAMs) are based on the bonding interaction between gold and sulfur and are a new type of organic ultra-thin membrane providing excellent performance. It is especially suitable for building a chemical and biological functional matrix membrane sensor due to its high order and stability [22], [23]. The mercaptoethanol in the mixed SAM is used as the diluent to control the concentration of biological molecules, such as antigens or antibodies, on the surface of the probe sensor. This fixes the immunocompetence and reduces the steric hindrance, which is favorable for an immune response and can effectively restrain non-specific adsorption of other miscellaneous protein biological molecules. Consequently, the practical application of the sensor has potential [24]–[27].

Previous applications of direct piezoelectric immunosensors for the diagnosis of *S. japonicum*-related diseases were conducted by Wu et al. [28]. A 32 kDa *S. japonicum* antigen was covalently attached to the crystal surface via a copolymer coating of hydroxyethyl methacrylate and methyl methacrylate. The *S. japonicum* antibody (*Sj*Ab) concentration was measured in the range of 7.2–90 µg ml^−1^. Nevertheless, this method does not sufficiently meet the sensitivity requirements for clinical analysis, particularly in the early stages of infection. In order to improve the sensitivity, amplified mass techniques using monoclonal antibodies have been shown to be an effective method with relatively high detection limits [17], [28], [29]. Later studies showed that *Sj*Ab concentrations can be linearly determined in the range of 10–200 ng ml^−1^ and that the determination limit is as low as 5 ng ml^−1^ using an amplification path based on an insoluble biocatalyzed precipitation product [30], [31]. Similar efforts to improve sensitivity were conducted by Wang et al. [32], who added polyethylene glycol to the buffer to enhance the immunoreaction. Recently, a piezoelectric immunosensor assay was developed with immobilizing immunoglobulin G (IgG) as a probe to detect circulating *S. japonicum* antigens (*Sj*Ag). This analytical strategy utilizes polyclonal antibodies with high specificity broad-spectrum recognition of a complex target. The linear dose–response relationship indicates that the systematic sensitivity of this method is below 150 Hz, and the lower limit of the detectable range is above 500 cercariae of *S. japonicum* infection for 2 weeks [33]. One of the principal advantages of immunosensors over other immunological techniques is their ability to be reused. The piezoelectric immunosensors in these studies, however, were either not regenerated, or the procedure was considered too complex.

In this paper, a simple, sensitive, and reusable piezoelectric immunosensor using hybrid SAMs for detection of *S. japonicum* was developed. The formation of highly ordered thiolate SAM on the crystal surface results in the immobilization of the antibody/antigen without altering the biological activity. This feature offers many advantages to the sensor performance, such as improvement in the binding activity of the antibody monolayer, increased sensitivity and reproducibility of the assay, ease of immunosensor regeneration, and prevention of nonspecific adsorption.

## Materials and Methods

### Ethics statement

All experiments involving rabbits were performed in accordance with protocols approved by the Animal Ethics Committee of South-Central University for Nationalities.

### Primary instruments

A rubber O-ring and plastic film was placed on one-side of the quartz crystal microbalance (QCM) to prevent it from touching liquid after the AT-cut gold-plated piezoelectric QCM (9 MHz) (Beijing Morningstar Company) was treated. Several additional instruments were used for this test, including a magnetic stirrer (Model JB-2, Shanghai Analytical Instrument Co. Ltd., Shanghai, China), a thermostat (Model CSS 501, Chongqing Experimental Equipment Factory, Chongqing, China) and a high frequency counter (Model FC 1250, Wellstar, Berlin, Germany). The TTCL-IC quartz oscillator circuit and testing pool were made in the Chemical Censing and Metrology Laboratory at Hunan University.

### Biological materials

Positive *Oncomelania hupensis* was supplied by the Institute of Yueyang Schistosomiasis Prevention and Treatment Center. New Zealand rabbits, at an average weight of 2500 g, were purchased from Hunan Agricultural University. This research was approved by the China Animal Protection Association. Normal rabbit serum (NRS), infected rabbit serum samples (IRS), and *S. japonicum* antigen were made in our laboratory according to previously published methods [34].

### Primary reagents

For tests conducted in this study, several reagents were used: Bovine Serum Albumin (BSA), mercaptopropionic acid (MPA), mercaptoethanol (ME), 1-Ethyl (3-dimethyl-aminopropyl)-Carbodiimide Imide Hydrochloride (EDC), N-hydroxy-succinimide (NHS), Polyethylene glycol (PEG, 4.0 kDa), made by Beijing Dingguo Biotechnology, FluKa Company, Sigma, and Tianjin Tiantai Chemical Company, respectively. Phosphate buffer solution (PBS, pH 7.0) was made using 0.067 M Na_2_HPO_4_ and 0.067 M KH_2_PO_4_, and Piranha lotion was made using 98% H_2_SO_4_ and 30% H_2_O_2_ at a ratio of 7∶3. All reagents were analytically purified, and double-distilled water was used.

### Mature *S. japonicum* soluble egg antigen (SEA) preparation

SEA was made according to methods reported in the literature [39]. Each rabbit was infected with 2500∼3 000 *S. japonicum* cercariae under local anesthesia and was euthanized 45 days later for liver collection and mature egg isolation. The samples were grinded with liquid nitrogen until there were no intact eggs under the microscope. The soluble protein was dissolved with the appropriate amount of PBS and oscillated for 12 h at 4°C in an EP tube. The supernatant was obtained after the protein was centrifuged at 12 000 rpm for 30 min, and the protein content was subsequently measured.

It is easy to form polymers when antibodies are preserved at −20°C or 4°C, especially with higher antibody concentrations (>2.0 mg ml^−1^). The polymers must be separated by 10 000 g centrifugation at 4°C for 30–60 minutes before being used.

### Rabbit anti-SEA antibodies

SEA was used to immunize the rabbits. One mg of sublimate SEA was diluted with normal saline and emulsified with the same volume of Complete Freund's Adjuvant (CFA). The mixed SEA was injected at multiple points in the back and hind leg muscles of the rabbits, and after 1–2 weeks, this process was repeated. After the second immunization, more injections were required to strengthen the immunization every 2–3 weeks. Blood was collected 7–14 days after each immunization, and the blood serum was separated to detect the immune effect. The carotid artery of the rabbit was exsanguinated when blood titers reached 1∶12800, measured by ELISA 5–7 days after the fourth immunization. Supernatant samples were taken after centrifugation at 4 000 rpm for 10 min followed by 1 h of depositing time at 37°C.

### SAM preparation

The quartz crystals were pretreated with Piranha solution (Note: the solution is strongly oxidizing upon preparation and should not be prepared until necessary). After rinsing with distilled water and drying, the pretreated crystals were immersed in a 1.0 mM MPA and 1.0 mM ME mixed solution at room temperature for ca. 5 h. The crystal was then washed with distilled water and air-dried. Finally, the self-assembly mixture of MPA and ME on the quartz crystal surface was ready.

### Antigen immobilization

Immobilization of *Sj*Ag was performed stepwise. Initially, a mixture of 30 µl 0.1 mg ml^−1^ EDC and 0.1 mg ml^−1^ NHS (volume ratio 1∶1) was dropped onto the surface of the quartz crystal with the mixed self-assembled modified monolayer gold electrode and incubated at room temperature for 30 min to activate the end of MPA carboxyl. The crystal was then washed with distilled water and PBS then air-dried. Subsequently, we dropped 30 ml of the *Sj*Ag diluent (volume ratio 1∶5 in PBS) onto the quartz crystal gold electrode surface followed by incubation at 37°C for 1 h, washing with PBS, and drying. Following the same steps described above, we continued by dropping 30 µl of a 10 mg ml^−1^ BSA and NRS mixture (volume ratio 1∶1) onto the crystal and incubation at 37°C for 1 h. This blocked non-specific binding sites on the surface of the crystal. Finally, the crystal was washed with PBS and air-dried for preservation.

### Determination

The crystal oscillator immobilized with *Sj*Ag was placed into the testing pool with 2.5 ml PBS mixed with PEG (pH 7.0, 3.5%) and NaCl (0.9%). Following agitation, 25 µl of each testing sample was added after the response frequency of the sensor was stable, and the change in frequency was quickly recorded. The corresponding antibody concentration was determined using a calibration curve according to the change of frequency.

### Sensor regeneration

After each test, the sensor was washed with an appropriate amount of 0.1 M glycine/hydrochloric acid (GHB, pH 2.3) for 15 min to dissociate *Sj*Ab on the surface. The sensor was then washed with PBS and air-dried before being used for the next sample.

## Results

### Mixed self-assembled film role

**Figure 1:**
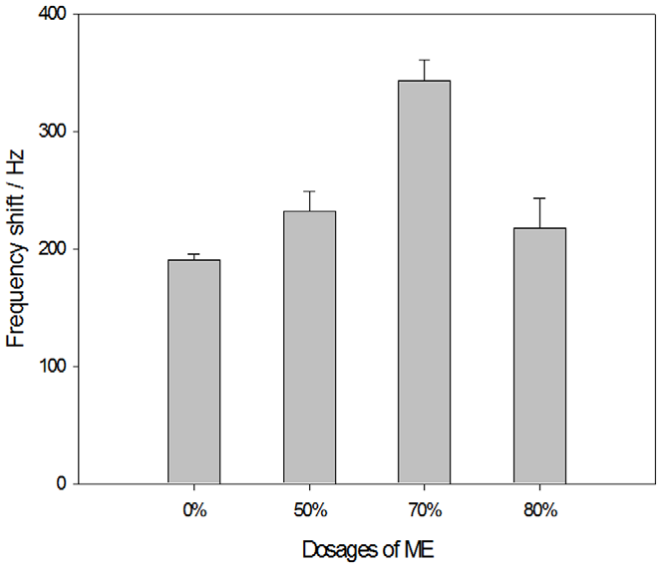
Effect of ME concentration in mixed SAM on *Sj*Ag immobilization. The best dose of ME in the mixed membrane was determined to be 70%.

The mixed SAM has two ends, which are made up of carboxyl compounds with different functional groups. The carboxyl at one end of the MPA carboxyl compound was used to covalently bond various immune biological molecules after activation by the coupling agent. The hydroxyl at one end of the ME sulfhydryl compounds functioned as a diluent in the immobilization of biological materials. The optimization of the amount of diluent in the membrane played a key role in controlling the concentration of biological molecules (antigen or antibody) on the surface of the sensor probe and in improving the performance of the detection method [35], [36]. Figure 1 shows the impact of the ME diluent content in the mixed film on the immobilization of *Sj*Ag. The response frequency of the sensor gradually increases with the proportion of ME, and the peak sensor frequency response occurs when the ratio reaches 70% (Figure 1). This indicates that, at this time, the immobilized *Sj*Ag molecules on the surface of the sensor are in good order and the steric hindrance**s** are small, which is indicative of an immune response. A decrease in the proportion of ME below 70% may be attributed to a lack of MPA in the mixed membrane leading to the inadequate *Sj*Ag immobilization on the surface of the sensor, which results in a decrease in the frequency. Therefore, a 70% dose of ME in the mixed membrane was selected for this experiment.

### Antigen dose optimization

**Figure 2:**
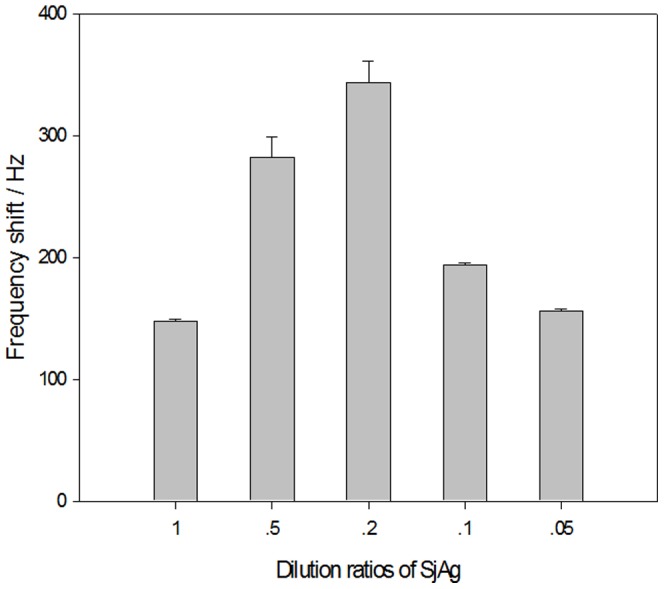
Effect of *Sj*Ag concentration on *Sj*Ag immobilization. The MPA and ME of volume ratios in mixed SAM are 3∶7. The *Sj*Ag dilution ratio is 1∶100. The best concentration of *Sj*Ag for immobilization is 0.2 mg ml^−1^.

The impact of the *Sj*Ag concentration (dilution ratio) on the immobilization of *Sj*Ag was evaluated in our experiments. The best immobilization concentration (dilution ratio) of *Sj*Ag is 0.2 mg ml^−1^, which results in the greatest frequency shift response value of the sensor (Figure 2). The frequency shift value decreases gradually with this concentration. As the active sites for *Sj*Ag immobilization on the sensor surface is limited, *Sj*Ag molecules in the mixed membrane reach a state of equilibrium when the concentration of *Sj*Ag increases to a certain value. However, when it exceeds this value, *Sj*Ag molecules are in disorder and the steric hindrance with *Sj*Ab increased. Therefore, the best concentration of *Sj*Ag was selected as 0.2 mg ml^−1^ for this experiment. In addition, the appropriate temperature conducive for improving the antigen-immobilization speed was 37°C for this experiment.

### Immune response optimization

According to the literature, different environmental media with different pH values [37] greatly affect antibodies of biological molecules and the spatial structure of antigens, which leads to differences in the forces between molecules. The most suitable range of pH for an immune response is usually 6.7–7.8, and the affinity between the antigens and antibodies is greatest when the pH of the solution is close to 7.0. For this reason, we used a PBS buffer with a pH of 7.0 in our experiments.

The ideal temperature for immune reactions was also investigated in this experiment. The results show that the sensors show the best performance of analysis and detection at room temperature (25°C). Therefore, all experiments were performed at room temperature (25°C).

**Figure 3:**
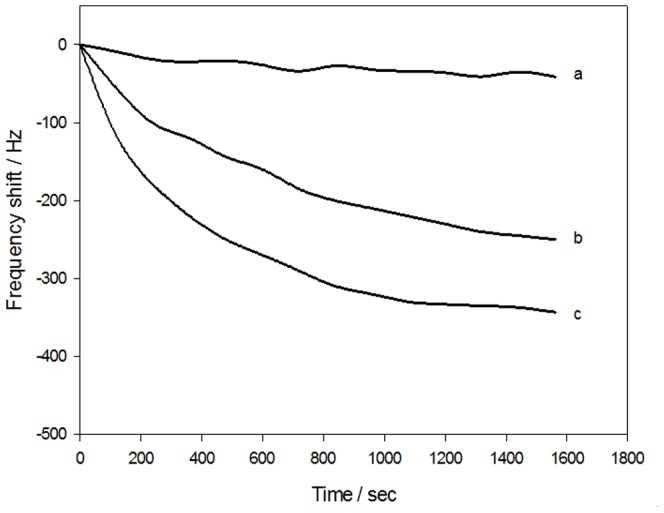
Typical real time frequency response characteristics of the immunosensor in PBS solution. (a) NRS as negative control with PEG; (b) *Sj*Ab without PEG (dilution ratio 1∶100); (c) *Sj*Ab with PEG (dilution ratio 1∶100). Using PEG can significantly enhance the sensor response signals. Immune response-mediated frequency shift values increased from 225 Hz (b) to 343 Hz (c). The corresponding time of immune response decreased from 1600 s (b) to 1200 s (c). Considering the analysis time and reproducibility, the time for both analysis and detection of the sensors in this experiment was 1200 s.

In addition, we used polyethylene glycol (PEG) as an accelerator of the immune response. PEG reduces the interaction between antibodies (or antigens) and water molecules and increases Coulomb forces between antibodies and antigens. PEG would decrease the hydration, strengthen the electrostatic attraction, and increase the entropy by randomization of formerly orientated water of hydration expelled into the bulk phase. This method has been applied successfully for a variety of biochemical analyses [38]–[40]. The potential enhancing effect of PEG on the immune response between *Sj*Ag and *Sj*Ab was also assessed in the experiment. Figure 3 shows experimental real time frequency response characteristics of the sensor for immune responses between samples with and without PEG. NRS was used as a negative control (Figure 3a). PEG can significantly enhance the response signal of the sensor (Figure 3). The results show that the immune response-mediated frequency shift value increased from 225 Hz (Figure 3b) to 343 Hz (Figure 3c), and the corresponding time for the completion of the immune response decreased from 1600 s (Figure 3b) to 1200 s (Figure 3c). Based on these results, we chose 1200 s as the time for sensor analysis and detection in this experiment.

### Closed experiment

**Figure 4:**
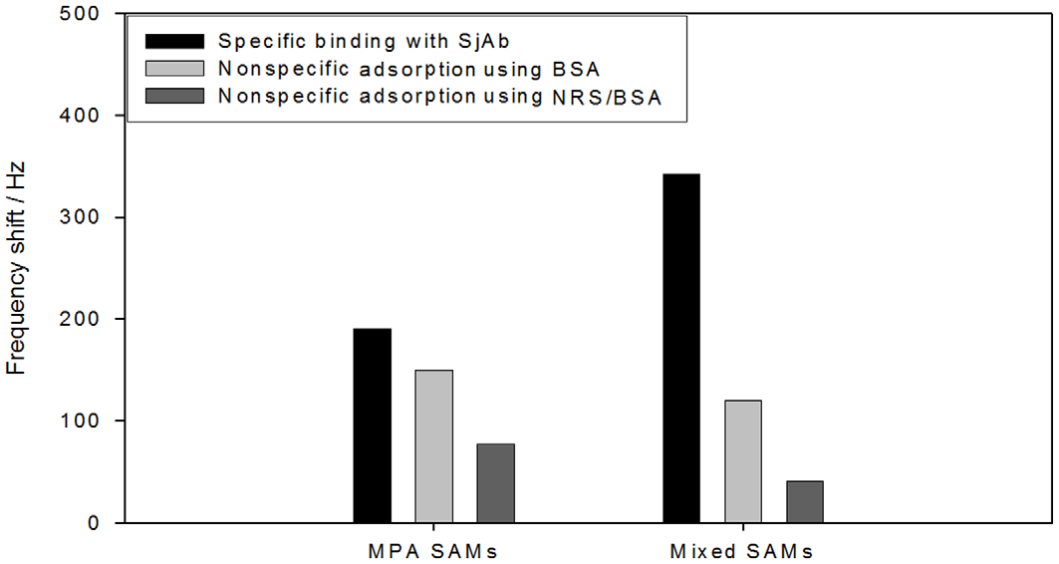
NRS frequency shift comparison between MPA - ME mixed SAM and MPA modifying sensors. *Sj*Ag and *Sj*Ab dilution ratios are 1∶5 and 1∶100 respectively. The frequency shift value using the sensor modified by MPA and ME hybrid films normalized to a blank (negative) NRS sample was significantly lower (120 Hz) than that of the sensor modified by MPA film and normalized to the NRS sample (156 Hz, with BSA used alone as a blocking reagent). However, the frequency shift values of the two sensors further declines to 41 Hz and 78 Hz, respectively, with the use of NRS/BSA mixture as blocking reagent.

One key issue to be resolved in the preparation and potential application of an immunosensor is how to effectively overcome non-specific absorption (background effect) in the samples. Research indicates that the “diluent” in mixed SAMs can not only control antigen (or antibodies) immobilization on the surface of the sensor, but can also effectively inhibit the non-specific adsorption of miscellaneous proteins or other biological molecules [24]–[27]. In addition, the NRS/BSA solution, used as the closure reagent, can block active sites on the surface of the sensor, which can effectively prevent cross-reactions and miscellaneous non-specific protein absorption [41]. Accordingly, *Sj*Ag bonded with a covalent coupling agent on the interface of the sensor, modified by MPA and ME mixed SAMs, and the remaining active sites on the surface were blocked with an NRS/BSA mixture to reduce non-specific absorption. The frequency shift value of the sensor with MPA only was used as a functional basement membrane, and a BSA blocking reagent was also compared (Figure 4). The frequency shift value of the sensor with the MPA and ME hybrid film normalized to a blank (negative) NRS sample (120 Hz) was significantly lower than that of the sensor with MPA film normalized to the NRS sample when BSA block was used alone (156 Hz) (Figure 4). However, the frequency shift values of the two sensors with the use of an NRS/BSA mixture as the blocking reagent further declined to 41 Hz and 78 Hz, respectively. The above results show that the combination of mixed SAMs and the NRS/BSA mixed double-inhibition reagent can effectively reduce the impact of non-specific adsorption on the surface of the sensor. In addition, Figure 4 indicates that the response frequency shift value of *Sj*Ab of the sensor using the MPA and ME hybrid film is much larger (343 Hz) than that of the sensor using the MPA monolayer (190 Hz).

### Primary response characteristics of sensors

**Figure 5:**
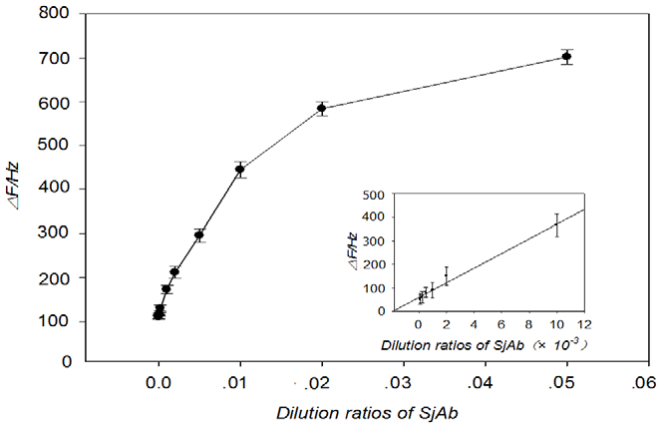
Calibration curves for the relationship between the frequency shift of immunoreaction and the dilution ratio of *Sj*Ab. The dynamic dilution range of *Sj*Ab is ∼1∶1500 to 1∶60 with the detection limit of ∼1∶1800 dilution, estimated according to the 3σ (standard deviation) rule. A sample with 1∶100 dilution of *Sj*Ab was determined repeatedly for five times. The average response frequency shift value was 340±19 Hz, and the relative standard deviation (RSD) among five runs was 9.1%.

The concentration (dilution ratio) of *Sj*Ab in the NRS samples was measured in PBS solution (pH 7.0, 3.5% PEG and 0.9% NaCl) using the developed immunosensor with the optimized experimental conditions described above. Figure 5 shows the calibration curve comparing response frequency shift values to the concentration of *Sj*Ab (dilution ratio). The linear range for *Sj*Ab detection by the sensor is from 1∶60 to 1∶1500 and the lowest limit of detection is 1∶1800, according to 3σ-Rules (standard deviation) (Figure 5). In addition, 5 parallel measurements with a known dilution ratio of the *Sj*Ab sample (1∶100) were taken with the sensor and resulted in an average response frequency shift value of 340±19 Hz and a relative standard deviation (RSD) of 9.1%. The results show that the immunosensor has a low limit of detection and good repeatability.

### Sensor regeneration

The ability to repeatedly use the sensor is an important indicator in determining its practicality [42]. Studies have shown that glycine can be effectively used to dissociate the antigen/antibody complexes from the surface of the crystal. In addition, glycine is a small molecule and can block amino binding sites on the surface of the probe to prevent non-specific adsorption of background proteins from the samples during recurrent testing [43]. A glycine/hydrochloric acid (GHB, pH 2.3) buffer solution was used as the eluent to renew the sensing probe in the experiment. The results show that renewing sensors in this way maintains high detection sensitivity after 4–5 uses, indicating that the sensor has a good regeneration performance. This may be attributed to the strong interactions of the gold and sulfur on the sulfhydryl mixed self-assembled film. Indeed, gold and sulfur bonds have high mechanical strength and chemical stability and can withstand the renewable impact of harsh reagents.

After the immunosensor is used 4 to 5 times, it should be cleaned with Piranha reagent to remove all organic material on the surface of the sensor to achieve complete regeneration. The sensor can then be used for the next round of experiments after re- immobilizing the antigen to its surface.

### Sample analysis

**Figure 6:**
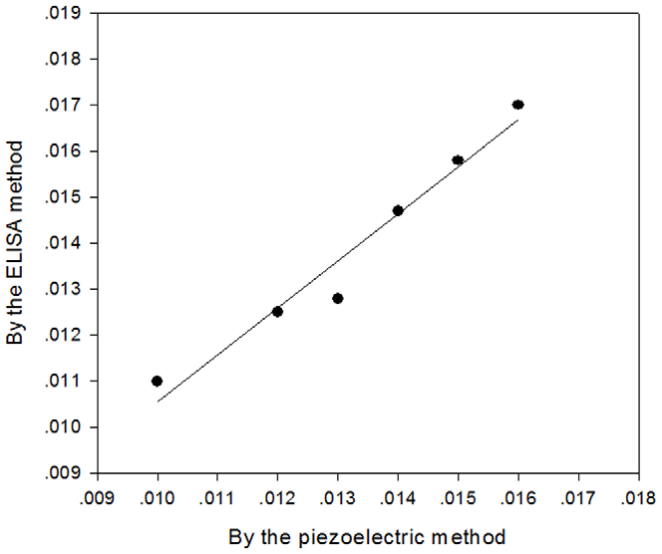
Linear regression analysis of immunosensors and ELISA methods. The correlation coefficient is 0.973.

Using the optimized experimental conditions described above, the concentration (dilution ratio) of *Sj*Ab in NRS samples was detected with the sensor to test its practical use. The association between the measured concentration and the calculated results were studied using the classic ELISA method [44] (Figure 6). We got a correlation coefficient 0.973 and a linear regression equation as follows:
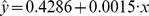



The results showed that our immune sensing technology may be used to evaluate the quality and quantity of the infected samples.

## Discussion

In this study, we present a new, reusable, simple, and sensitive piezoelectric immunosensor, which incorporates a sulfhydryl mixed SAM as a functional basement membrane for the efficient covalent immobilization of *Sj*Ag to directly measure *Sj*Ab in NRS.

The four main findings are presents in this study. First, this fixes the immunocompetence and reduces the steric hindrance, which is favorable for an immune response. Second, the combination of a mixed SAM and an NRS/BSA mixed reagent can effectively reduce non-specific absorption on the surface of the sensor. Third, PEG, was used as an immunoreaction enhancer to increase the response signal, which improved sensitivity (signal) and detection limits considerably. The last, the glycine/hydrochloric acid buffer solution is used as the eluent to renew the sensing probe and this method of regeneration is both stable and reproducible.

Because of their simplicity, low cost, and real-time response, SAM piezoelectric quartz crystal sensors are gaining attention as competitive tools for various fields [45]–[47]. However, the use of quartz crystals is impracticable for multi-detection. Researchers have been actively seeking alternatives by using other piezoelectric materials or ferroelectric piezoelectric materials, such as piezoelectric zirconate titanate (PZT). On the other hand, the sensitivity using quartz crystals still remains lower than what has been achieved by other immunosensor devices. To meet the requirements of clinical laboratories, such as detecting early-stage infections, further studies are in progress to improve the QCM immunosensor sensitivity.

In conclusion, we believe that the system described in this study is a potential alternative to current diagnostic methods for *Schistosomiasis japonicum*-mediated desease. Importantly, it may be applicable both in the clinical laboratory or used this portable device outside the laboratory. With the development of chemical and new materials technology, the method of the molecular immobilization on the membrane will be updated, which will promote the technique of piezoelectric immunesensors rapidly. This technique can also be applied for additional molecular immunoassays, and the research on sensor array which can detect multiple samples at the same time is a direction of the piezoelectric immunesensors in the future.
